# Effects of Rolling Parameters on Stress–Strain Fields and Texture Evolution in Al–Cu–Sc Alloy Sheets

**DOI:** 10.3390/ma18235414

**Published:** 2025-12-01

**Authors:** Guoge Zhang, Lijie Liu, Tuo Li, Shan Tang, Bo Gao

**Affiliations:** 1 State Key Laboratory of Structural Analysis, Optimization and CAE Software for Industrial Equipment, Department of Enginering Mechanics, Dalian University of Technology, Dalian 116023, China; zhangguoge@mail.dlut.edu.cn (G.Z.); 201972025@mail.dlut.edu.cn (L.L.); shantang@dlut.edu.cn (S.T.); 2 School of Materials Science and Engineering, Taiyuan University of Science and Technology, Taiyuan 030024, China; lituo@tyust.edu.cn; 3 International Research Center for Computational Mechanics, Dalian University of Technology, Dalian 116023, China

**Keywords:** Al–Cu–Sc alloy, rolling speed, feeding rate, pass schedule, stress–strain fields, rolling force, texture evolution

## Abstract

This work examines how rolling speed, feeding rate, and pass schedule—with a constant total reduction—affect the stress–strain fields, rolling force, and texture evolution of Al–Cu–Sc alloy sheets. A coupled finite element (FEM) and viscoplastic self-consistent (VPSC) framework is employed and compared with EBSD measurements to connect macroscopic fields with microscale texture changes. Results indicate that increasing rolling speed raises the effective strain rate and deformation heating, which lowers peak rolling force and improves in-plane stress homogenization on the RD–ND plane, while enhancing surface–core incompatibility and residual-stress gradients along the ND–TD direction. A higher feeding rate mainly intensifies work hardening, slightly elevates rolling force, and promotes near-surface stress/strain localization; in contrast, multi-pass schedules redistribute deformation between passes and reduce macroscopic stress concentration. Texture analyses show a speed-induced rotation from 〈001〉 toward 〈111〉 orientations, strengthening shear-related components; KAM maps suggest increased local orientation gradients consistent with higher stored energy. The simulations capture the principal experimental trends across conditions, supporting the use of the combined framework for trend-level process guidance. Overall, the findings clarify parameter–microstructure relationships and provide a basis for designing rolling routes that balance force reduction, stress uniformity, and texture control in Al–Cu–Sc sheets.

## 1. Introduction

Aluminum alloys are widely used for sheet products because of their high specific strength, corrosion resistance, and thermal conductivity [1,2,3]. Among them, Al–Cu–Sc alloys have attracted increasing attention for lightweight structural applications in aerospace and transportation, owing to the microalloying effect of scandium (Sc) [4,5]. The addition of Sc promotes the formation of coherent Al_3_Sc nano-precipitates, which are among the most effective strengthening phases in aluminum alloys. The foundational role of Al_3_Sc in imparting significant strength via precipitation hardening has been well–established in binary Al–Sc systems [6,7]. Furthermore, these precipitates are highly valued for their exceptional ability to inhibit recrystallization and stabilize fine-grained structures [8,9] their remarkable thermal stability governed by coherency loss kinetics [10,11], and their demonstrated potential in complex alloys like Al–Cu to simultaneously enhance strength and corrosion resistance [12,13,14,15,16]. Rolling is the principal route to produce thin and wide sheets from such alloys; hence, process parameters directly shape microstructure and final properties [17,18,19,20,21]. In particular, rolling speed, feeding rate, and pass schedule define the local strain rate and metal flow pattern. Even with a fixed total reduction, different parameter combinations can alter the stress–strain field, residual stresses, rolling force, and texture evolution [22,23,24,25,26,27,28,29].

Despite extensive studies on aluminum sheet rolling, two aspects remain insufficiently resolved for Al–Cu–Sc sheets with the practical constraint of constant total reduction. First, most prior works examine single-factor influences or emphasize macroscopic properties and static recrystallization, while the partitioning of deformation among passes and its consequences for inter-pass stress/strain accumulation, through-thickness residual-stress gradients, and force fluctuations have not been systematically quantified [30,31]. Second, the links between parameter-controlled strain-rate histories and microscale pathways—grain rotation, dislocation storage, and the emergence of shear-related texture components—are often discussed qualitatively, with limited multi-scale evidence that connects fields to texture evolution under comparable processing paths [32,33]. Representative studies on high-strength Al alloys report strong sensitivities of texture to pass schedule and reduction strategy [34,35], but a coordinated analysis that separates parameter effects from the total-reduction effect and couples macro fields to microtexture for Al–Cu–Sc is still scarce.

Recent advances in modeling offer tools to address these gaps. Finite element method (FEM) simulations can resolve the evolution of stress/strain and rolling force under specified process paths, while viscoplastic self-consistent (VPSC) models capture polycrystalline reorientation under rate-sensitive slip [36,37,38,39]. However, predictive use of a combined FEM–VPSC framework—benchmarked by EBSD texture measurements under identical conditions—remains challenging for Al–Cu–Sc due to the sensitivity of responses to strain-rate gradients, frictional shear, and inter-pass history.

Motivated by these observations, this study investigates how rolling speed, feeding rate, and number of passes—with a fixed total reduction—govern the macro- and micro-scale evolution of Al–Cu–Sc sheet rolling. We couple FEM (for stress/strain fields and rolling force) with VPSC (for texture development) and validate the trends against EBSD measurements on the same processing paths. The central questions are: (i) how parameter choices rebalance in-plane stress homogenization on the RD–ND plane against through-thickness gradients on the ND–TD plane; (ii) how strain-rate histories relate to grain rotation toward shear-related components and local orientation gradients; and (iii) to what extent multi-pass schedules mitigate macroscopic stress concentration via inter-pass relaxation.

The contributions are threefold. First, we delineate parameter-specific roles with constant total reduction, decoupling the influence of rolling speed, feeding rate, and pass schedule on stress/strain distributions and residual stresses. Second, we establish a multi-scale correspondence between macroscopic fields and microtexture evolution, clarifying how rate effects promote 〈111〉-related components and local misorientation signatures. Third, we provide a calibrated FEM–VPSC workflow, trend-validated by EBSD, that can serve as a reference for parameter screening and texture control in Al–Cu–Sc sheet rolling (trend-level guidance rather than point predictions).

## 2. Simulation and Experimental Methods

### 2.1. Simulation Model Development

**Figure 1:**
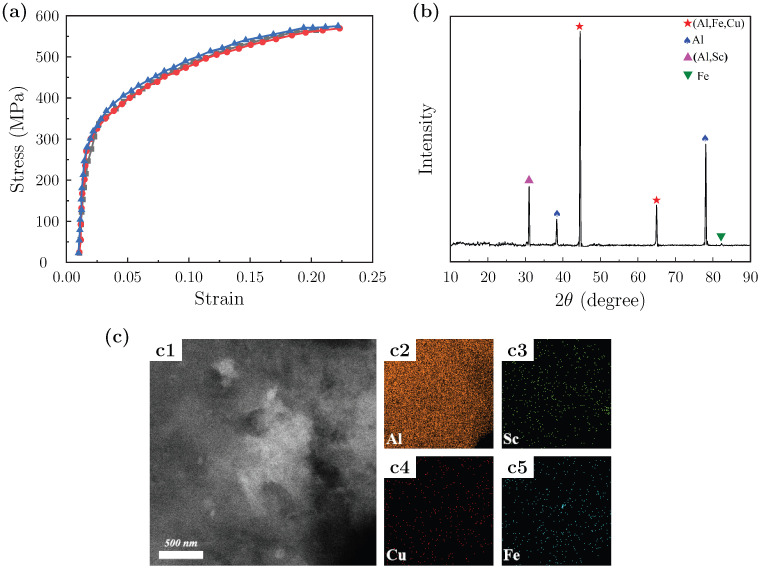
(**a**) Room-temperature true stress–strain curve of the Al–Cu–Sc alloy sheet used for constitutive modeling; (**b**) XRD patterns of the RD–ND cross-section; (**c**) Microstructure and elemental distribution of the initial slab. (**c1**) HAADF-STEM image; (**c2**–**c5**) Elemental distribution maps.

Finite element simulations of the rolling process were performed using ABAQUS 2017. A rigid–plastic framework was adopted to approximate the high plastic strain conditions of sheet rolling. The room-temperature true stress–strain curve used for the constitutive description of the Al–Cu–Sc alloy and the XRD pattern of the RD–ND cross-section are shown in Figure 1a,b, respectively. The microstructure and elemental distribution of the initial slab are presented in Figure 1c, where panel c1 shows the HAADF-STEM image and panels c2–c5 display the elemental maps of the main constituents.

The workpiece, with initial dimensions of 150 mm × 80 mm × 10 mm, was discretized into 100,000 C3D8R elements (8-node linear brick, reduced integration) to ensure sufficient resolution of local stress and strain gradients. The rolling rolls were modeled as analytical rigid bodies, and their interaction with the sheet was described using a surface-to-surface contact algorithm. A constant Coulomb friction coefficient of 0.1 was applied based on typical values reported for cold-rolling processes [40,41].

**Figure 2:**
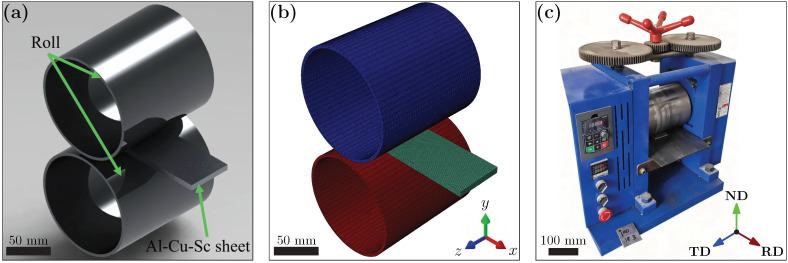
Finite element simulation and experimental setup: (**a**) rolling mill model; (**b**) finite element mesh scheme; (**c**) laboratory rolling setup.

The overall simulation configuration—including the finite element mesh, and its correspondence to the experimental setup—is illustrated in Figure 2a–c. Table 1 lists the basic physical properties of the alloy used in both simulation and experiments. Table 2 summarizes the chemical composition of the Al–Cu–Sc Alloy.

### 2.2. Experimental Procedure

A commercial Al–Cu–Sc alloy with the properties listed in Table 1 was selected for experimental verification. It was homogenized at 500 °C for 24 h using an SG-XL1400 box furnace, followed by direct water quenching to retain the homogeneous microstructure. This treatment was intended to achieve a uniform initial microstructure and minimize element segregation, which can significantly reduce creep life [43]. The homogenized billets were then machined into sheets with dimensions of 150 mm × 80 mm × 10 mm ± 0.5 mm using a DK7735 wire electrical discharge machine (EDM). Surface grinding was performed to ensure consistent surface finish and minimize surface defects before rolling. The TEM images of the alloy in this initial state are presented in Figure 1c.

Cold rolling was conducted at room temperature on a two-high rolling mill with a roll diameter of 250 mm ± 0.2 mm and a roll length of 300 mm ± 0.2 mm. Rolling experiments were performed under different process parameters (rolling speed, feeding rate, and number of passes) while maintaining a constant total reduction of 40%, corresponding to a final sheet thickness of 6 mm. The rolls are made of 9Cr_2_Mo high-carbon chromium-molybdenum alloy steel, utilizing a surface quenching process to enhance body hardness and form a hardened layer for improved fatigue resistance. This experimental design ensured that the influence of individual parameters could be decoupled from the total deformation effect.

The evolution of stress–strain fields, residual stress distribution, and microtexture was characterized at different rolling stages. Measurements were taken on two principal sections: the RD–ND (rolling–normal) plane, which reflects in-plane deformation and stress homogenization, and the ND–TD (normal–transverse) plane, which captures through-thickness stress gradients and texture evolution. These results were subsequently compared with the simulation predictions for validation and further analysis.

## 3. Results and Discussion

### 3.1. Stress Analysis

Rolling of the Al–Cu–Sc alloy sheet is a continuous, multi-stage deformation process involving the entry, deformation, and exit stages. The evolution of the stress field across these stages directly affects plastic flow behavior, residual stress distribution, and ultimately the microstructure and properties of the final sheet. Three key process parameters—feeding rate (linear entry speed of the sheet), rolling speed (angular velocity of the rolls), and number of passes—play a decisive role in shaping the stress state during rolling. These parameters influence contact stress at the entry stage, plastic flow stress during deformation, and residual stress in the final product. Understanding their effects is therefore essential for clarifying the physical mechanisms of deformation and for establishing process–structure correlations [44,45].

#### 3.1.1. Stress Analysis at Entry Stage: Effects of Rolling Speed and Feeding Rate

**Figure 3:**
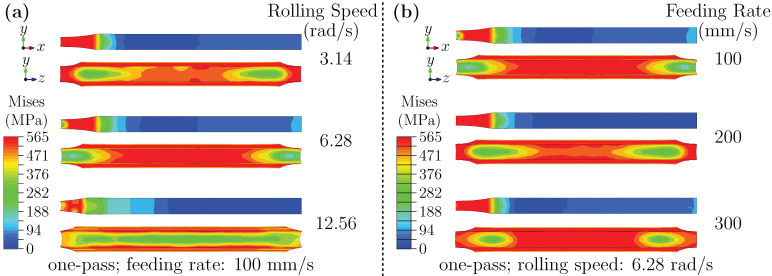
(**a**) Stress distribution during the entry stage under different rolling speeds; (**b**) Stress distribution during the entry stage under different feeding rates.

Figure 3a presents the simulated stress distributions on the RD–ND (rolling–normal) and ND–TD (normal–transverse) planes under three representative rolling speeds: 3.14 rad/s (low), 6.28 rad/s (medium), and 12.56 rad/s (high), with the feeding rate fixed at 100 mm/s. Minor fluctuations (within ± 0.1%) inherent to the motor control system are negligible, given the order-of-magnitude differences between the selected speeds, and do not affect the observed trends. At low rolling speed, high and localized stress is observed in the roll-sheet contact zone, while the ND–TD plane shows a gradient decreasing from the surface toward the core. As the speed increases to a medium level, the stress distribution broadens, and edge regions exhibit more pronounced gradients. At high rolling speed, the overall stress on the RD–ND plane becomes more symmetric through the sheet thickness, accompanied by a distinct gradient along the rolling direction. In all cases, displacement in the rolling direction is greater on the outer surface than in the core, and this difference increases with rolling speed.

These observations suggest that strain rate and deformation heating jointly affect stress evolution. In hot-working studies, such coupled strain-rate/temperature sensitivities are often interpreted through processing-map concepts; although a full processing-map construction is not applicable to room-temperature cold rolling, the same governing variables provide a useful mechanistic reference. Here, rolling speed primarily modulates the local effective strain rate and its accompanying deformation heating, thereby shifting the balance between work hardening and rate/thermal softening and controlling flow heterogeneity. At a lower rolling speed, the slower dissipation of energy tends to result in more localized stress concentrations. Medium rolling speed appears to accentuate edge effects. For a higher speed, the interplay between deformation heating and dynamic softening contributes to a more symmetric stress profile, while also enhancing the difference between surface and core regions [46,47].

Figure 3b shows the influence of feeding rate (100, 200, 300 mm/s) on the entry-stage stress field at a fixed rolling speed of 6.28 rad/s. A lower feeding rate produces a more pronounced ND–TD stress gradient near the edges and a stress field on the RD–ND plane that extends forward and backward relative to the roll bite zone. Stress magnitude generally decreases from the surface to the core. The elevated stress concentration at low feeding rate is possibly related to higher local strain rates and intensified dislocation activity [48]. Moreover, slower feeding exacerbates deformation inhomogeneity, further accentuating edge stresses. The observed surface-to-core stress decay is likely related to the combination of shear deformation near the surface and the triaxial compressive state in the core. Additionally, the stress field ahead of the roll gap is influenced by restricted metal flow and evolving deformation-zone geometry.

#### 3.1.2. Stress Analysis at Deformation Stage: Effects of Rolling Speed and Feeding Rate

**Figure 4:**
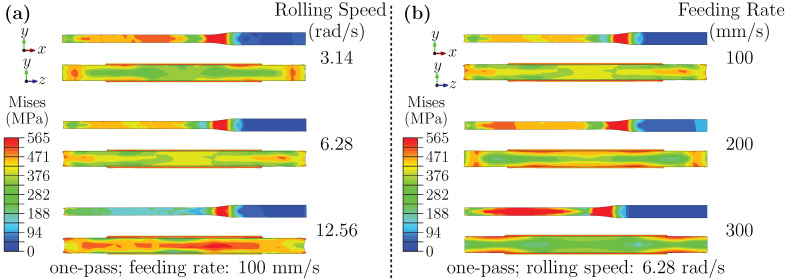
(**a**) Stress distribution during the deformation stage under different rolling speeds; (**b**) Stress distribution during the deformation stage under different feeding rates.

Figure 4a shows the stress distributions during the deformation stage under different rolling speeds. As rolling speed increases, the stress distribution on the RD–ND plane becomes more uniform, while the peak stress decreases. In contrast, the ND–TD plane exhibits an opposite trend. At low speed, stress is mainly concentrated near the sheet edges, with a peak value of approximately 519.8 MPa. At medium speed, the high-stress zone moves outward, and the peak drops to about 378.3 MPa. At high speed, stress concentration shifts toward the core, forming a gradient that decreases radially outward and significantly reducing edge stresses.

This transition may be associated with strain-rate effects and thermal–mechanical interactions. At higher speeds, increased strain rates accompanied by rapid surface cooling may restrict plastic flow near the surface, potentially elevating triaxial stresses in the core. In contrast, low-speed rolling allows more uniform deformation but induces edge stress concentration, likely due to limited lateral flow. Additionally, variations in contact arc length and deformation zone geometry could also play a role in the observed stress evolution.

Figure 4b illustrates the effect of feeding rate during deformation. Increasing the feeding rate leads to higher stresses on the RD–ND plane but lower stresses in the ND–TD core region. This redistribution suggests an enhanced deformation mismatch between the surface and core regions. An increased feeding rate raises the strain rate, which is accommodated microscopically by an accelerated multiplication of dislocations. Furthermore, the presence of Al_3_Sc precipitates aggravates dislocation pile-up, enhancing the work hardening and higher overall stress on the RD–ND plane. On the ND–TD plane, stress concentration at low feeding rate is primarily attributed to poor deformation compatibility near the free edges. With increasing the feeding rate, a pronounced metal flow difference develops between the surface and core, enhancing deformation inhomogeneity. This shifts stress localization to the outer surface, while the core remains in a lower-stress state. These interpretations are qualitative and may depend on friction conditions and local temperature rise.

#### 3.1.3. Effect of Pass Number: Inter-Pass Relaxation and Stress Redistribution

**Figure 5:**
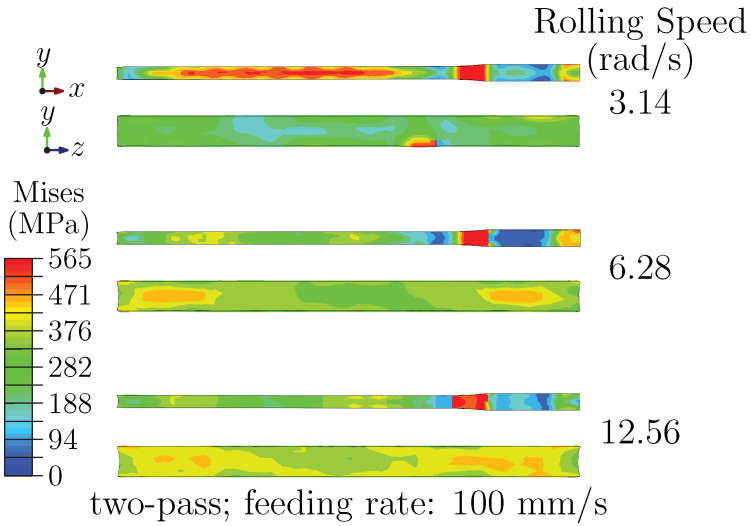
Stress distribution during two-pass rolling at different rolling speeds.

The number of passes also significantly influences stress evolution. As shown in Figure 5, two-pass rolling reduces stress concentration compared with a single-pass process. This reduction is associated with dividing the total 40% reduction into two stages, which allows partial stress relaxation between passes and mitigates concentrated material flow [49,50]. Furthermore, the microstructural response underlines the benefit of multi-pass rolling. The presence of Al_3_Sc precipitates in Al–Cu–Sc alloy is accompanied by the formation of extensive dislocation pile-ups, as revealed by microstructural characterization. Under single-pass deformation, these microstructural features correspond to localized high-stress regions. Multi-pass processing promotes a more homogeneous microstructure through enhanced dynamic recovery, effectively alleviating the stress concentration.

At higher rolling speed, strain-rate gradients along the sheet width become more pronounced, amplifying edge effects and increasing stress concentration points on the ND–TD plane. Nevertheless, the more homogeneous deformation achieved with two passes suppresses localized stress aggregation and results in a more dispersed stress distribution overall.

#### 3.1.4. Stress Analysis at Exit Stage: Coupled Effects of Rolling Speed, Feeding Rate, and Pass Number

**Figure 6:**
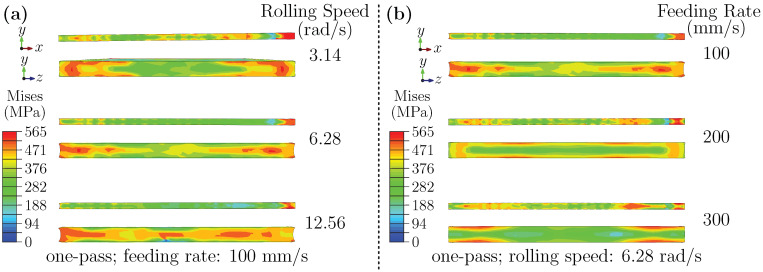
(**a**) Stress distribution at the exit stage under different rolling speeds; (**b**) Stress distribution at the exit stage under different feeding rates.

Figure 6a summarizes the exit-stage stress distributions under different rolling speeds. Increasing speed is associated with reduced stress concentration on the RD–ND plane and a more evident multi-zone incompatibility on the ND–TD plane, which appears as an arc-shaped gradient from surface to core. A plausible explanation is that shorter roll–sheet contact time at higher speeds intensifies near-surface shear and accumulates sufficient surface strain, while the core experiences delayed and less extensive deformation. In parallel, stronger width-wise strain-rate gradients at higher speeds impose greater flow constraints at the edges than at the center, further contributing to the observed through-thickness gradient. Upon cooling, differential contraction between the more-deformed surface and the less-deformed core may preserve part of this gradient.

Figure 6b shows the effect of feeding rate on the exit-stage stress field. On the ND–TD plane, higher feeding rate tend to shift stress concentration from the core toward the surface and to weaken the arcuate pattern, whereas on the RD–ND plane, stresses become more dispersed and symmetric. These tendencies are consistent with the combined influence of strain-rate sensitivity and deformation compatibility: lower feeding rate allows deeper stress penetration due to longer deformation times, while higher rates can increase near-surface softening and promote a more uniform in-plane distribution.

**Figure 7:**
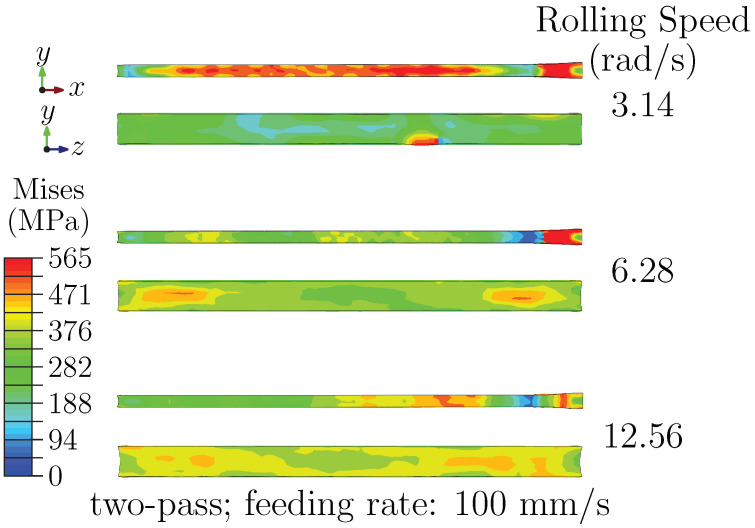
Stress distribution at the exit stage under two-pass rolling.

The influence of pass number is presented in Figure 7. For two-pass schedules, increasing rolling speed still reduces the peak stress on the RD–ND plane and promotes a more uniform pattern. On the ND–TD plane, the distribution evolves from localized near-surface concentration at low speed to a clearer outward-increasing gradient at higher speed.

This evolution suggests a combined role of inter-pass strain redistribution (which mitigates macroscopic concentration) and strain-rate gradients (which promote a microscopic through-thickness imbalance). Partial recovery between passes may influence the final gradient, possibly by relaxing accumulated strain in the mid-thickness while preserving surface-layer deformation.

#### 3.1.5. Equivalent Stress Evolution During Rolling

**Figure 8:**
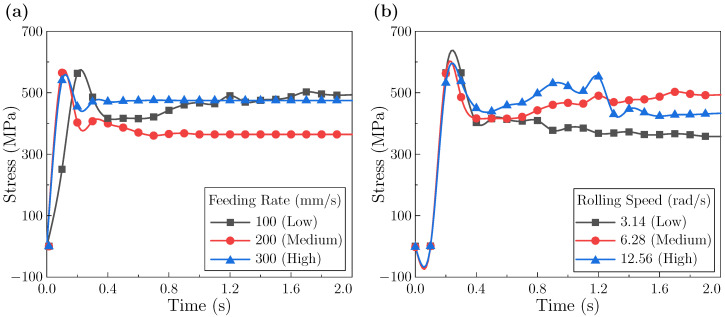
Equivalent stress at the leading edge of the sheet: (**a**) under different feeding rates; (**b**) under different rolling speeds.

Figure 8 reports the equivalent stress at the sheet leading edge. As shown in Figure 8a, the feeding rate noticeably affects the temporal profile. At a low feeding rate, the stress exhibits a “continuous rise → stabilization” trend with hysteresis. The longer contact time likely permits more complete plastic flow and sustained dislocation activity, allowing work hardening to develop until a quasi-balance is reached. At higher feeding rate, peak stresses are comparable, whereas the stabilized level differs: the medium rate stabilizes around ∼364 MPa with small oscillations, while the high rate remains near ∼474 MPa. The higher stabilized level at the fast rate may reflect a less effective softening relative to dislocation accumulation [51,52], whereas the medium rate appears to achieve a more balanced hardening–softening state.

Figure 8b shows that the leading-edge stress versus time under different rolling speeds generally follows an “increase → decrease → stabilization” pattern. Three features are noted: (i) the low-speed case exhibits the highest peak, which is suggestive of limited deformation heating combined with more pronounced work hardening; (ii) the medium-speed case has a slightly higher peak than the high-speed case, a trend potentially reflecting the more dominant role of thermal softening at the highest speed; and (iii) during intermediate stages, the stress level tends to increase with speed, possibly linked to accelerated dislocation accumulation under non-steady-state conditions.

**Figure 9:**
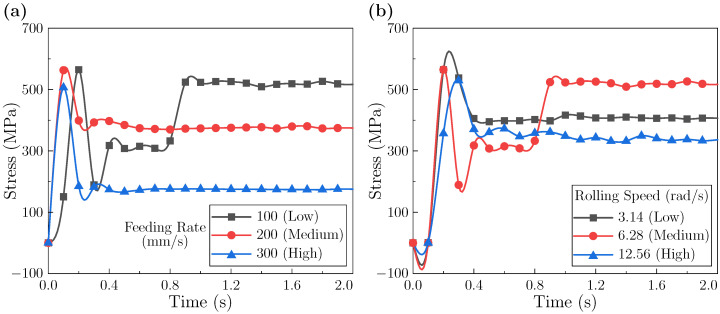
Equivalent stress at the leading edge within the sheet interior during rolling: (**a**) under different feeding rates; (**b**) under different rolling speeds.

The interior leading-edge response is shown in Figure 9. In Figure 9a, the low feeding rate produces a pronounced peak followed by a rapid drop and secondary surges before stabilization. This multi-peak behavior likely reflects repeated episodes of constrained flow and subsequent coordination as the edge continuously enters the deformation zone. In contrast, medium and high feeding rate show a single peak, a drop, and a smoother stabilization. The stabilized stress decreases with feeding rate: ∼518 MPa (low), ∼373 MPa (medium), and ∼175 MPa (high), consistent with shorter accumulation times and faster establishment of mechanical equilibrium at higher rates.

As shown in Figure 9b, increasing rolling speed lowers the peak stress (low > medium > high). The stabilized stress, however, follows medium (∼520 MPa) > low (∼403 MPa) > high (∼333 MPa). This divergence suggests that peak stress is governed by the time available for accumulation, whereas stabilization reflects how quickly coordinated flow is established. High speed appears to favor rapid coordination and thus a lower final level; low speed adapts gradually, yielding a moderate level; the medium case may be fast enough to limit accumulation but not fast enough to fully coordinate, resulting in the highest stabilized value.

These stress evolution patterns provide a crucial macro-scale context for the subsequent microstructural analysis. The observed transition from localized surface stresses at low rolling speed to more symmetric distributions and stronger through-thickness gradients at higher speeds is expected to influence grain rotation behavior and the activation of shear-related texture components, which will be examined in Section 4.

### 3.2. Strain Analysis

#### 3.2.1. Strain Analysis at Entry Stage: Effects of Rolling Speed and Feeding Rate

**Figure 10:**
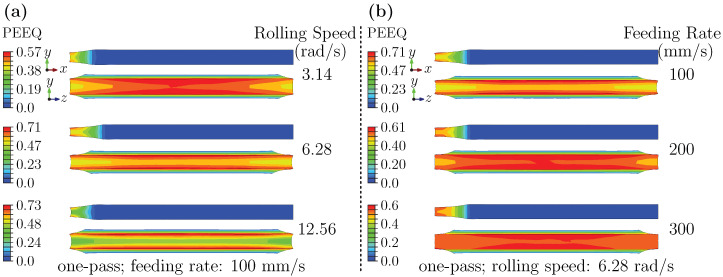
Equivalent plastic strain distribution at entry: (**a**) varying rolling speeds; (**b**) varying feeding rates.

Figure 10 shows the equivalent plastic strain (PEEQ) distributions on the RD–ND and ND–TD planes during entry. Increasing rolling speed and decreasing feeding rate are associated with higher peak PEEQ and a more pronounced through-thickness gradient on the ND–TD plane. In all cases, the strain consistently decreases from the core to the surfaces along the TD. Additionally, the edge regions of the upper and lower surfaces exhibit higher metal flow, forming arc-shaped high-strain zones along both sides. These features are consistent with strain-rate-dependent plasticity: elevated effective strain rates intensify dislocation multiplication and concentrate deformation, while reduced constraints at the edges and differential friction between the rolls promote the characteristic arc-shaped pattern.

#### 3.2.2. Strain Analysis at Deformation Stage: Effects of Rolling Speed, Feeding Rate, and Pass Schedule

**Figure 11:**
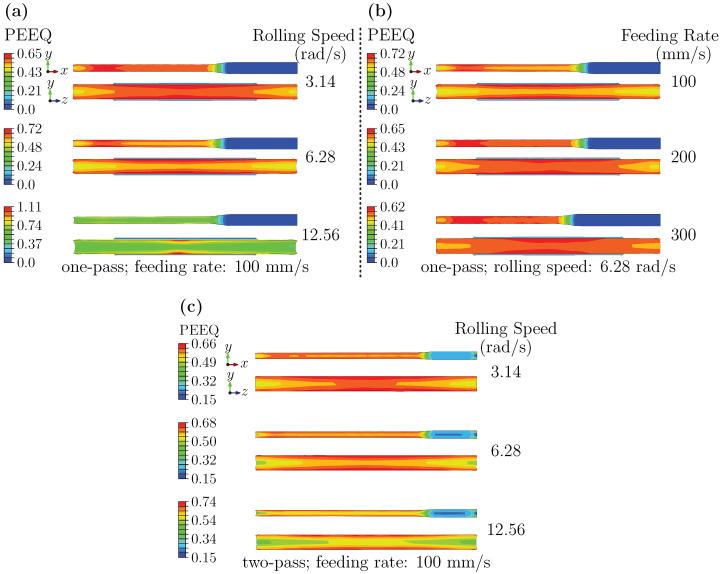
PEEQ distribution during forming: (**a**) different rolling speeds; (**b**) different feeding rates; (**c**) two-pass schedules at different rolling speeds.

Figure 11 summarizes the forming-stage PEEQ distributions. In all conditions, a gradient from the surface toward the core is observed. Figure 11a indicates that increasing rolling speed elevates peak strain (approximately doubling from low to high) while promoting a more uniform distribution on the RD–ND plane. Figure 11b shows that higher feeding rate also increase the strain rate but yield comparatively lower peaks and more uniform in-plane distributions; however, edge arc-shaped localization becomes more apparent. Figure 11c suggests that a two-pass schedule lowers peak strain to roughly half that of a single pass while maintaining the monotonic decrease from surface to core along the ND.

These results appear to be rationalized by the combined action of strain rate and pass schedule. Higher rolling speed, associated with increased strain rates, are linked to greater dislocation accumulation and more intense plastic flow. This correlates with a rise in peak strain and a tendency toward greater in-plane uniformity. Higher feeding rates distribute deformation over shorter times, which seems to constrain peak accumulation yet preserve surface-dominated shear, amplifying edge localization. Multi-pass rolling divides total reduction and introduces inter-pass relaxation, allowing partial annihilation of dislocations and consequently reducing peak strain.

#### 3.2.3. Strain Analysis at Final Product Stage: Dependence on Rolling Speed and Feeding Rate

**Figure 12:**
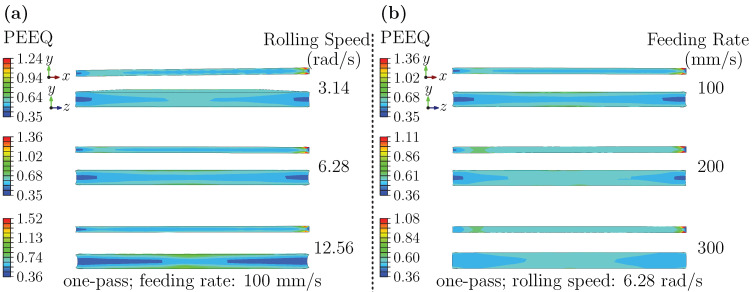
PEEQ in the final product: (**a**) different rolling speeds; (**b**) different feeding rates.

Figure 12 presents the final-stage PEEQ distributions. Peak PEEQ at this stage exceeds that of the entry and forming stages. On the ND–TD plane, strain gradually decreases from the center toward both sides along the TD. The outer-surface strain exceeds that of the inner surface along the ND, though their difference is smaller than in earlier stages. On the RD–ND plane, the inner–outer difference increases with rolling speed; at low speed, the maximum strain tends to appear near the core. Figure 12b further indicates that along the TD, there exists a qualitative correlation between high-strain regions and higher residual stress. The outer surface strain increases with the feeding rate.

These observed patterns align with cumulative strain over multiple passes and the inhomogeneity associated with the final deformation. The higher peak strain appears to reflect the combined effect of strain accumulation and heightened deformation resistance during the final pass. The center-to-edge gradient on the ND–TD plane is compatible with constrained transverse flow in the center and relatively freer deformation near the edges. Higher speeds can promote homogenization and reduce inner–outer differences, potentially aided by deformation heating and dynamic softening, whereas higher feeding rates intensify surface shear and frictional effects, making outer-surface localization more evident. The qualitative overlap of high PEEQ and higher residual stress is consistent with strain-gradient-induced internal stress and local work hardening.

### 3.3. Rolling Force Analysis

#### 3.3.1. Effect of Rolling Speed

**Figure 13:**
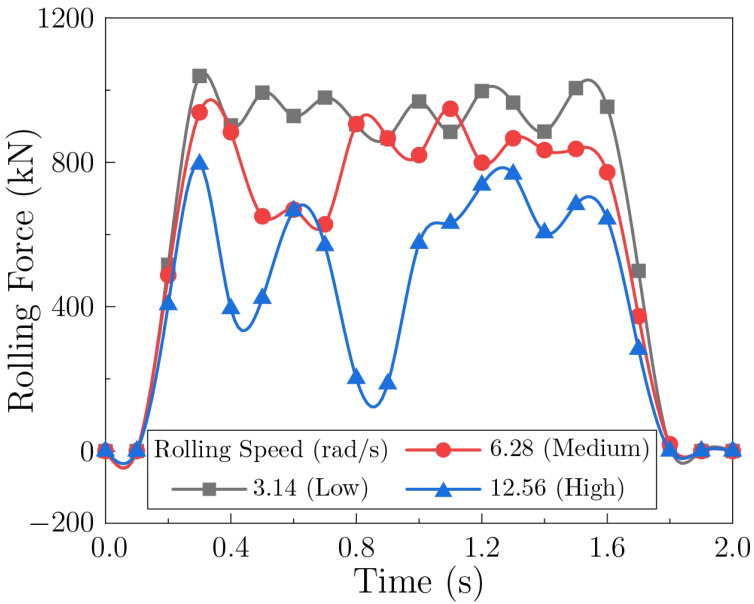
Rolling force during sheet forming under different rolling speeds.

Figure 13 shows the rolling-force histories for different rolling speed. Force fluctuations appear in all cases; the amplitude is smallest at low speed and becomes more pronounced as speed increases. Meanwhile, the average rolling force decreases with speed, with the high-speed condition showing the lowest overall level.

A possible explanation for these observations may lie in the varying deformation mechanisms activated at different rolling speeds. Under low-speed conditions, the deformation is likely to proceed more uniformly, promoting a closer balance between dislocation multiplication and dynamic recovery, which would contribute to the smaller stress fluctuations observed. With increasing speed, the associated rise in strain rate could encourage faster yet less homogeneous dislocation accumulation, potentially giving rise to transient instabilities in the flow stress [51,53]. Furthermore, the deformation heat generated at higher speeds may not be dissipated completely within the short roll-sheet contact time, tending to reduce the average flow stress through thermal softening. Changes in lubrication conditions and the potential onset of vibration at elevated speeds may also play a role in the increased fluctuation amplitude.

#### 3.3.2. Effect of Feeding Rate

**Figure 14:**
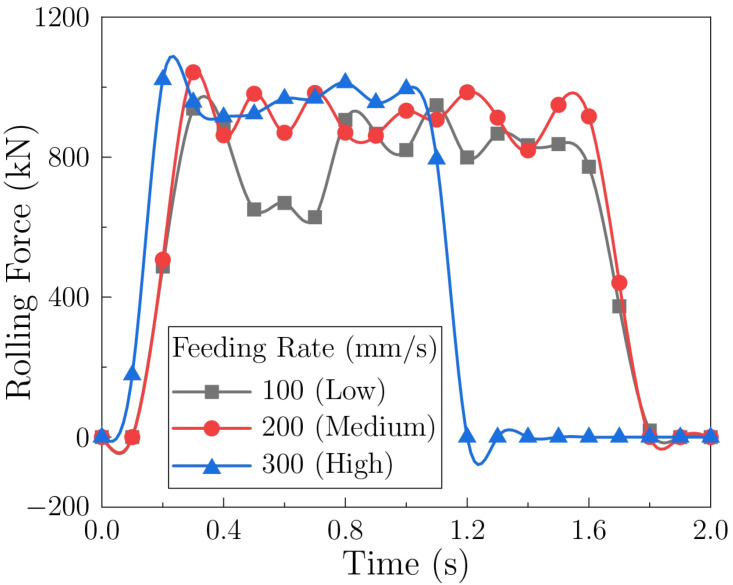
Rolling force during sheet forming under different feeding rates.

Figure 14 presents the force histories for different feeding rates. With increasing rate, the peak rolling force shows a slight upward trend, while fluctuation amplitude decreases. The modest increase in peak force is consistent with higher strain rates and the strengthening effect of precipitates, which impede dislocation motion [54,55]. The reduced fluctuation amplitude at higher rates may be attributed to a more stable and continuous metal supply into the deformation zone and a more uniform shear state at the roll–sheet interface.

#### 3.3.3. Effect of Differential Speed Ratio

**Figure 15:**
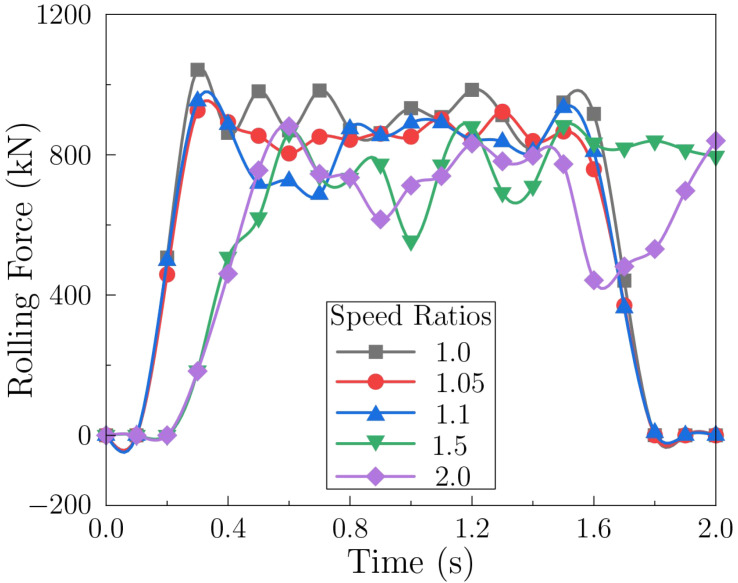
Rolling force under different differential speed ratios.

Figure 15 illustrates the influence of differential speed ratio (base speed 6.28 rad/s). Synchronous rolling (ratio 1.0) produces a higher rolling force than asynchronous conditions. As the ratio increases, the rolling force decreases and reaches a minimum of ∼460 kN at a ratio of 2.0, compared with ∼797 kN for synchronous rolling.

The reduction under asynchronous rolling may be related to additional through-thickness shear arising from roll-speed mismatch, which is generally considered to facilitate plastic flow and lowers deformation resistance. A larger ratio enhances this shear and further reduces the force. However, asynchronous rolling also introduces a shear-stress gradient decreasing from the fast-roll side to the slow-roll side, which may drive sheet warping toward the slow-roll side. This tendency persists even at higher reductions because it originates from the intrinsic velocity differential. Consequently, differential speed ratios should be selected by balancing force reduction with forming quality (flatness, microstructural uniformity, and surface integrity), while controlling shear strain and residual stress.

In summary, the combined analysis of stress, strain, and rolling force reveals parameter-dependent deformation modes that reshape both in-plane and through-thickness field distributions. These field characteristics—such as enhanced surface shear, strain-rate-driven gradients, and inter-pass relaxation—are expected to directly affect grain rotation pathways, texture intensities, and local lattice curvature. Mechanistically, the effects of rolling parameters are primarily mediated by their control of the local effective strain rate and friction-assisted shear in the deformation zone. Higher rolling speed or feeding rate elevates the strain rate and deformation heating, shifting the balance between dislocation multiplication/storage and rate/thermal softening, which explains the observed changes in rolling force and stress/strain heterogeneity. In Al–Cu–Sc sheets, stable Al_3_Sc nano-precipitates further pin dislocations and suppress recovery, promoting strain-rate-dependent dislocation storage (consistent with increased KAM) and facilitating grain rotation toward shear-related 〈111〉 components.

Although a systematic post-rolling mechanical-property evaluation is beyond the scope of this paper, the present field and texture results imply clear trend-level property consequences. Specifically, higher rolling speed and feeding rate elevate the effective strain rate, promoting dislocation storage (consistent with higher KAM) and stronger shear-related 〈111〉 components, which are expected to enhance strength/work hardening but may also intensify through-thickness residual-stress gradients and anisotropy. In contrast, multi-pass schedules mitigate stress/strain localization via inter-pass relaxation, implying improved deformation uniformity and potentially more stable formability. These qualitative links provide a process–structure basis for future property-focused studies. Section 4 builds on these findings by examining the corresponding microstructural signatures through EBSD characterization and VPSC simulations.

## 4. Experiment and Texture Analysis

This section examines texture evolution during rolling using Electron Backscatter Diffraction (EBSD) and compares the measurements with predictions from a viscoplastic self-consistent (VPSC) model. By correlating these microstructural results with the macroscopic field patterns described in Section 3—such as stress homogenization, through-thickness gradients, and rolling-force fluctuations—we aim to establish a trend-level correspondence between process parameters and crystallographic reorientation. The objective is twofold: (i) to characterize orientation distributions and local lattice curvature under different rolling speeds, and (ii) to assess whether the coupled simulation framework reproduces the main experimental trends in a parameter-consistent manner.

### 4.1. EBSD Measurement Setup and Results of IPF and KAM Analysis

The EBSD observations provide direct microstructural evidence for the macro–micro linkages hypothesized in Section 3. Variations in stress distribution and deformation heterogeneity translate into distinct orientation trends and dislocation patterns, allowing a detailed examination of how processing parameters influence the internal structure.

**Figure 16:**
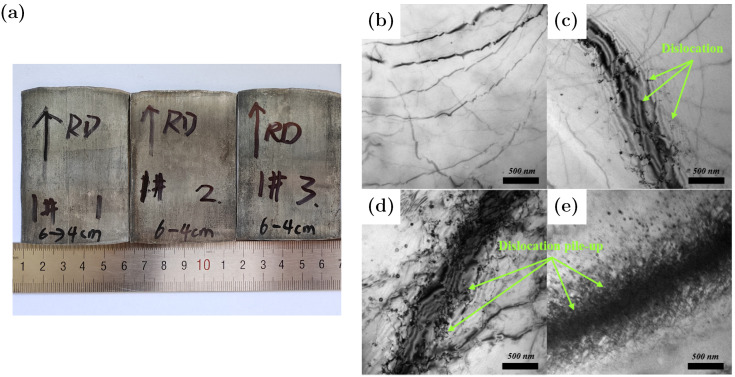
(**a**) Sheets prepared under different rolling speeds; (**b**) TEM image of the sample prior to rolling; (**c**) low speed; (**d**) medium speed; (**e**) high speed.

Figure 16a shows representative sheets prepared at different rolling speeds. EBSD measurements were performed on the RD–ND plane to capture through-thickness and in-plane orientation features under the same preparation protocol. To improve indexing quality and reduce electron-beam scattering, the sample tilt was set to 70 °, the working distance to 20 mm, and the accelerating voltage to 20 kV. A step size of 1.5 μm and a nominal magnification of 800× were adopted. A 500 × 500 μm central area was selected to minimize edge effects and to provide comparable deformation histories across samples [56]. These settings balance spatial resolution with scan efficiency and are sufficient for resolving grain-scale orientation gradients in the present alloy.

In the initial state (Figure 16b), dislocations are nearly absent. After rolling (Figure 16c–e), plastic deformation induces substantial dislocation multiplication, resulting in a remarkable increase in dislocation density compared to the initial condition. Regarding the effect of rolling speed: at a low speed (Figure 16c), moderate dislocation proliferation occurs, forming relatively loose tangles; at a medium speed (Figure 16d), dislocation tangles become denser and more complex; at a high speed (Figure 16e), the dislocation density peaks, exhibiting extremely dense dislocation accumulation. In summary, the dislocation density in the initial state is considerably lower than that in all rolled samples. For the rolled specimens, the dislocation density progressively increases with the rolling speed, which is attributed to the enhanced plastic deformation and the consequent promotion of dislocation generation and pile-up under higher strain rates.

**Figure 17:**
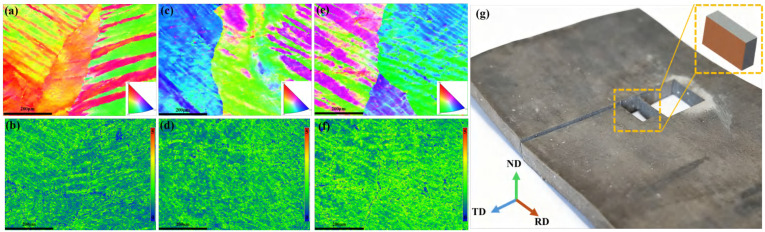
IPF and KAM maps under different rolling speeds: (**a**,**b**) low; (**c**,**d**) medium; (**e**,**f**) high; (**g**) Schematic diagram of the RD–ND cross-section for sampling characterization.

Figure 17 presents the Inverse Pole Figure (IPF) maps and Kernel Average Misorientation (KAM) maps obtained at low, medium, and high rolling speed. Under low-speed conditions, orientations are dominated by 〈101〉 and 〈001〉, with a comparatively lower fraction of 〈111〉-oriented grains. As the rolling speed increases, the fraction of 〈111〉-oriented grains gradually rises while 〈001〉 decreases, indicating a rotation tendency toward 〈111〉/〈101〉-related components. At high speed, 〈001〉 further diminishes, 〈101〉 slightly declines, and transition orientations between 〈001〉 and 〈111〉 become more prevalent.

KAM maps reveal a concurrent increase in local orientation gradients with speed, suggesting higher geometrically necessary dislocation (GND) content and more heterogeneous micro-strain at elevated rates. This observation is consistent with the notion that increased strain rate enhances dislocation multiplication relative to dynamic recovery, thereby raising stored energy and potentially influencing subsequent recrystallization kinetics [57,58,59,60]. It should be noted that KAM provides a qualitative indicator of local lattice curvature; its interpretation as a direct measure of dislocation density is approximate and may depend on step size and noise filtering.

These microstructural signatures are consistent with the field-level findings: higher rolling speed, which were shown to enhance surface shear and amplify through-thickness gradients, correspond to a pronounced increase in 〈111〉-oriented grains and elevated KAM values, reflecting intensified dislocation storage and shear activity.

### 4.2. Comparison Between VPSC Predictions and Experimental Results

To quantify texture evolution under the tested conditions, a VPSC model was employed. The initial texture was represented by 6000 discrete grain orientations, imported as the starting ODF sample. Grain reorientation was computed using crystal plasticity constitutive relations consistent with the rolling kinematics, and the same processing path used in the experiments was applied in the simulations to ensure comparability [36,37,61]. The model focuses on rate-dependent slip-dominated plasticity; dynamic softening mechanisms are not explicitly introduced, which is noted when interpreting residual discrepancies.

**Figure 18:**
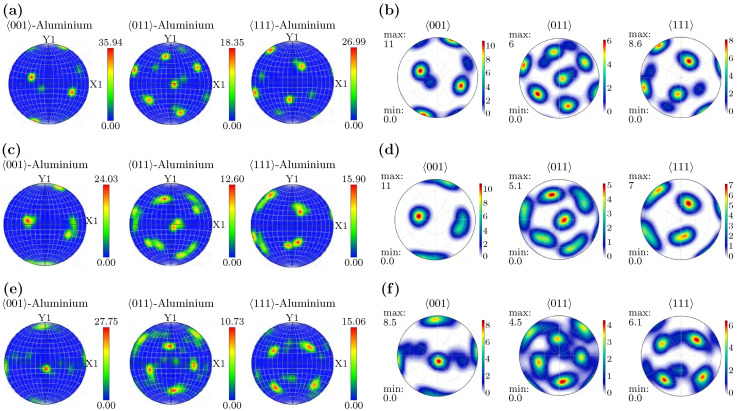
Experimental (**left**) and VPSC-simulated (**right**) pole figures at different rolling speeds: (**a**,**b**) low; (**c**,**d**) medium; (**e**,**f**) high.

Figure 18 compares experimental pole figures with VPSC results for the same speed conditions. At low speed, the measured texture exhibits medium strength with relatively concentrated components and clear contours; the simulation captures the main distribution trend with slightly stronger local concentration. At medium speed, overall intensity and symmetry increase in both experiment and simulation, and the agreement in coverage and intensity gradients is the most apparent among the three conditions. At high speed, the measured texture becomes more diffuse with lower overall intensity; the simulation reproduces the diffuse trend but shows somewhat higher component intensities.

Overall, the VPSC model reproduces the principal evolution tendencies across speed, indicating that the rate-sensitive slip-based framework is suitable for describing the dominant reorientation paths under the present conditions. Residual differences—particularly the overprediction of component intensity at high speed—may be linked to the absence of explicit dynamic softening and/or subgrain rotation mechanisms in the current implementation, as well as to experimental factors such as surface preparation, step size, and the finite sampling of the ODF.

In summary, EBSD measurements and VPSC simulations reveal consistent trends in texture evolution across different rolling speeds. This agreement supports the reliability of the combined FEM–VPSC–EBSD framework for capturing rate-sensitive orientation changes in Al–Cu–Sc sheets. Parallel trends are observed in both macroscopic deformation fields (Section 3) and the microstructural response (Section 4). Specifically, parameter-induced changes in strain rate and stress distribution are systematically mirrored in the crystallographic reorientation. This macro–micro consistency underscores the framework’s utility for analyzing and designing rolling processes across multiple scales. Minor discrepancies are also noted, highlighting the potential roles of dynamic softening and microstructural heterogeneity. These aspects can be systematically addressed in future work by extending the experimental protocol and refining the constitutive assumptions in the model.

## 5. Conclusions

This study systematically investigated the influence of rolling process parameters on the macro- and micro-scale responses of Al–Cu–Sc alloy sheets with a constant total reduction. By integrating finite element simulations (FEM), viscoplastic self-consistent (VPSC) modeling, and EBSD characterization, the work successfully validates the proposed contributions by linking process conditions to field evolution, texture development, and deformation mechanisms. The main conclusions are as follows:

1. **Macroscopic field evolution is dominated by strain-rate effects.** Rolling speed plays the most critical role in shaping stress and strain distributions. Higher speeds elevate strain rates, leading to a reduction in peak rolling force and improved stress uniformity on the RD–ND plane. At the same time, they amplify surface–core deformation incompatibility, resulting in residual stress gradients along the ND–TD direction. Feeding rate primarily influences dislocation multiplication and work hardening, thereby raising stress levels and shifting concentration toward the surface. Multi-pass rolling redistributes deformation across passes and facilitates partial recovery, promoting stress homogenization.
2. **Process parameter interactions govern rolling force behavior and energy demand.** Increased rolling speed enhances rolling force fluctuations, while higher feeding rate slightly raise the peak load but stabilize the process by improving flow continuity. The solution for force reduction is demonstrated through asynchronous rolling, which reduces deformation resistance by up to 30% at a differential speed ratio of 2.0. However, the accompanying asymmetric shear can cause sheet warping, underscoring the importance of balancing force reduction with dimensional accuracy and forming quality.
3. **Microstructural evolution is highly sensitive to deformation kinetics.** EBSD and VPSC analyses reveal that higher rolling speed strengthen shear deformation contributions, promoting grain rotation toward 〈111〉 orientations and shifting texture toward shear-dominated components. Elevated strain rates also increase dislocation density, manifested as enhanced local orientation gradients. These observations demonstrate that process parameters—especially rolling speed—can be used as effective levers for tuning texture, anisotropy, and recrystallization behavior.
4. **Integrated simulation–experiment approaches enable predictive process design.** The combined FEM–VPSC framework reproduces key macroscopic and microscopic trends across parameter conditions, with close agreement to EBSD measurements. This capability enables the isolation and quantification of parameter effects, providing a foundation for virtual process design, rapid parameter screening, and reduced reliance on extensive experimental iterations.

It is important to note that this study focuses on the forming characteristics and microstructural evolution during cold rolling, rather than the final mechanical properties of the processed sheet. The primary contribution lies in elucidating the relationship between rolling parameters and the resulting stress-strain distributions, which provides a critical foundation for understanding the subsequent mechanical performance.

## Figures and Tables

**Table 1:** Physical properties of the Al–Cu–Sc alloy [13,42].

Density (g·cm^−3^)	Young’s Modulus (GPa)	Poisson’s Ratio (—)	Thermal Conductivity (W/(m·K))	Yield Strength (MPa)
2.85	73	0.33	173	325

**Table 2:** Content of main elements in Al–Cu–Sc Alloy (%).

P	Sn	Sc	Ca	Fe	Cu	Al
<0.001	<0.001	0.009	0.0011	0.012	2.69	Bal.

## Data Availability

The raw data supporting the conclusions of this article will be made available by the authors on request.

## Abbreviations

The following abbreviations are used in this manuscript:

C3D8R	8-node linear brick element with reduced integration
EBSD	Electron Backscatter Diffraction
EDM	Electrical Discharge Machining
FEM	Finite Element Method
IPF	Inverse Pole Figure
KAM	Kernel Average Misorientation
PEEQ	Equivalent Plastic Strain
RD	Rolling Direction
ND	Normal Direction
TD	Transverse Direction
VPSC	Viscoplastic Self-Consistent (model)
